# Improved performance of an intensive care unit after changing the admission triage model

**DOI:** 10.1038/s41598-023-44184-6

**Published:** 2023-10-09

**Authors:** Alexandre S. Larangeira, Ana Luiza Mezzaroba, Fernanda K. Morakami, Lucienne T. Q. Cardoso, Tiemi Matsuo, Cintia M. C. Grion

**Affiliations:** 1 Intensive Care Division, Londrina State University, Londrina, Brazil; 2 Internal Medicine Department, Londrina State University, Rua Robert Koch 60, Vila Operária, Londrina, Paraná 86038-440 Brazil; 3 Statistics Department, Londrina State University, Londrina, Brazil

**Keywords:** Epidemiology, Risk factors

## Abstract

The aim of this study is to analyze the effect of implementing a prioritization triage model for admission to an intensive care unit on the outcome of critically ill patients. Retrospective longitudinal study of adult patients admitted to the Intensive Care Unit (ICU) carried out from January 2013 to December 2017. The primary outcome considered was vital status at hospital discharge. Patients were divided into period 1 (chronological triage) during the years 2013 and 2014 and period 2 (prioritization triage) during the years 2015–2017. A total of 1227 patients in period 1 and 2056 in period 2 were analyzed. Patients admitted in period 2 were older (59.8 years) compared to period 1 (57.3 years; *p* < 0.001) with less chronic diseases (13.6% vs. 19.2%; *p* = 0.001), and higher median APACHE II score (21.0 vs. 18.0; *p* < 0.001)) and TISS 28 score (28.0 vs. 27.0; *p* < 0.001). In period 2, patients tended to stay in the ICU for a shorter time (8.5 ± 11.8 days) compared to period 1 (9.6 ± 16.0 days; *p* = 0.060) and had lower mortality at ICU (32.8% vs. 36.9%; *p* = 0.016) and hospital discharge (44.2% vs. 47.8%; *p* = 0.041). The change in the triage model from a chronological model to a prioritization model resulted in improvement in the performance of the ICU and reduction in the hospital mortality rate.

## Introduction

The limitation of resources for health care is a well-known scenario. With regard to access to specialized beds in intensive care, there is often no availability for all the people who need it. To optimize available resources, it is part of an intensivist's duties to make decisions about priorities for access to intensive care units (ICU)^1^.

When the available resources are supplanted by the demand for a particular service, a service queue is formed, that is, repressed demand. To understand the reasons for the formation of this queue, one can refer to the queuing theory. Queuing theory is an area of the exact sciences that studies the probability of queue formation. The queuing process is composed of three elements: arrival regime, service regime, and queue discipline. The queue discipline refers to the rules that define the order given to the patients who will be treated. For this, there are several possibilities, such as first-come, first-served care, random care, or priority for certain categories of patients^2^.

The most logical and most applied intuitive model in everyday life is the chronological one, that is, the first to arrive is the first to be served. However, in the health system and in situations of high repressed demand for ICU beds, this model can have serious consequences, especially for patients with greater chances of recovery. Waiting for an ICU bed may reduce these chances of survival as some treatments are available exclusively within the ICU environment^3^.

A triage system must be as precise clinical as possible. Slightly permissive triage is preferable, but in a heavily overloaded system it may not be feasible, especially in situations of high demand for beds. If triage is overly restrictive, it may be associated with increased mortality. To decide on admission to an ICU, there are several models to be considered^4–7^. Among these is the ICU admission prioritization model, in which the discipline of the waiting list follows a structured triage system. A task force of the North American Society of Critical Care Medicine proposed a triage system with five levels of priority, where level 1 is the highest priority for ICU admission and level 5 is the lowest priority^5^.

The aim of the current study is to analyze the effect of implementing a prioritization and triage model for intensive care unit admission compared to a chronological model on the outcome of critically ill patients.

## Materials and methods

This study was reviewed and approved by the University Hospital of Londrina Institutional Review Board (IRB)/ethics committee, Approval Number: 3.377.114, approval date: 07/JUN/2019, study title: “The effect of changing the screening model for admission to the intensive care unit in the outcome of critically ill patients”. Procedures were followed in accordance with the ethical standards of the institutional responsible committee on human experimentation and with the Helsinki Declaration of 1975. For this study informed consent has been waived by University Hospital of Londrina Institutional review board (IRB)/ethics committee due to the anonymity and retrospective nature of the study.

Retrospective longitudinal study carried out by convenience sampling of adult patients admitted to the Intensive Care Unit (ICU) of a University Hospital from January 2013 to December 2017. This is a retrospective analysis of prospective collected data from all patients admitted to the ICU during the study period. This period was chosen because it contains complete data of patients admitted to the ICU in accordance with the institutional protocol at the time. Patients under 18 years of age, patients with an ICU length of stay of less than 24 h, and ICU readmissions during the same hospital stay were excluded from the analysis. The primary outcome considered was vital status at hospital discharge.

The general data collected for all ICU admissions were age, sex, date of hospital and ICU admission, primary diagnosis of ICU admission, sector of origin, date of ICU and hospital discharge, outcome at ICU and hospital discharge.

Information on the presence of chronic diseases, need for mechanical ventilation, need for dialysis, use of vasoactive drugs, and scores on the *Acute Physiology and Chronic Health Evaluation* II (APACHE II)^8^, *Sequential Organ Failure Assessment* (SOFA)^9^, and *Therapeutic Intervention Scoring System 28* (TISS 28)^10^ were also collected on the first day of ICU admission. Other important variables collected were length of hospital stay and length of stay in the ICU.

Patients were included in the study according to 2 periods. Period 1 included the years 2013 and 2014 and in this period the triage for admission to the ICU was in chronological order. Period 2 included the years 2015–2017 and the triage for ICU admission followed the order of priorities^5^.

Until the end of 2014, the institutional protocol for triage and admission to the ICU was chronological (first come, first treated)^7^. In the context of high demand for intensive care, this hospital changed the form of triage to a prioritization model for ICU admission based on the guidelines of the Society of Critical Care Medicine^5,11^. The triage was performed by the physician on duty from the rapid response team who evaluates all requests for admission to the ICU, according to the institutional protocol. This model is based on the classification of patients with demand for intensive care into five groups, denominated:

- Priority 1: Critically ill patients who require life support for organ failure, intensive monitoring, and therapies only provided in the ICU environment.
- Priority 2: Critically ill patients who require life support for organ failure, with a significantly lower probability of recovery and who would like to receive intensive care therapies but not cardiopulmonary resuscitation in case of cardiac arrest.
- Priority 3: Patients with organ dysfunction who require intensive monitoring and/or therapies.
- Priority 4: Patients with organ dysfunction who require intensive monitoring and/or therapies but with a lower probability of recovery/survival.
- Priority 5: Terminal or moribund patients with no possibility of recovery; such patients are in general not appropriate for ICU admission (unless they are potential organ donors).

### Statistical analysis

Continuous variables are expressed as mean and standard deviation (SD) or median and interquartile range (ITQ) according to data distribution. Categorical variables are expressed as proportions. Descriptive statistics were used for the presentation of all relevant variables. Data are presented in graphs and tables. The nonparametric Mann–Whitney test was used to compare continuous variables. Categorical variables were compared using Pearson’s chi-square or Fisher's exact test and the chi-square test for linear trend. Bivariate analysis was performed to analyze the association of screening with study variables. Cox regression analysis was applied to assess factors that contributed independently to explain the hospital outcome, and the effect of each factor was expressed as a proportional hazard ratio (HR) and 95% confidence interval (95% CI). The cumulative risk of in-hospital mortality was described by analyzing the survival curve obtained by Cox regression. Sample size power was not calculated. The significance level used was 5% and the analyses were performed using SPSS Statistics for Windows (IBM Corp. Released 2010. IBM SPSS Statistics for Windows, Version 19.0. Armonk, NY: IBM Corp.).

### Ethics approval and consent to participate

This study was reviewed and approved by the University Hospital of Londrina Institutional Review Board (IRB)/ethics committee, Approval Number: 3.377.114, approval date: 07/JUN/2019, study title: “The effect of changing the screening model for admission to the intensive care unit in the outcome of critically ill patients”. Procedures were followed in accordance with the ethical standards of the institutional responsible committee on human experimentation and with the Helsinki Declaration of 1975. For this study informed consent has been waived by University Hospital of Londrina Institutional review board (IRB)/ethics committee due to the anonymity and retrospective nature of the study.

### Consent for publication

All authors consent for publication of the manuscript.

## Results

During the study period, 3644 patients were admitted to the intensive care beds of the research institution. We excluded 69 patients under 18 years of age, 164 patients readmitted to the ICU during the same hospital stay, and 128 for a length of stay in the ICU of less than 24 h. Thus, a total of 3283 patients were analyzed, of whom 1227 were admitted in period 1 and 2056 in period 2.

Table 1 presents the comparison of clinical characteristics at ICU admission and outcomes according to the study periods. Patients admitted in period 2 were older (59.8 years) compared to period 1 (57.3 years; *p* < 0.001) with less chronic diseases (13.6% vs. 19.2%; *p* = 0.001), and higher median APACHE II score (21.0 vs. 18.0; *p* < 0.001)) and TISS 28 score (28.0 vs. 27.0; *p* < 0.001). In period 2, patients tended to stay in the ICU for a shorter time (8.5 ± 11.8 days) compared to period 1 (9.6 ± 16.0 days; *p* = 0.060) and had lower mortality at ICU (32.8% vs. 36.9%; *p* = 0.016) and hospital discharge (44.2% vs. 47.8%; *p* = 0.041). The diagnosis of sepsis on ICU admission was more frequent in the second period of the study.

**Table 1:** Clinical characteristics and outcomes according to the study period.

Variable	Period 1 (n = 1227)	Period 2 (n = 2056)	Total (n = 3283)	*p* value
Age				< 0.001*
Mean ± SD	57.3 ± 19.0	59.8 ± 18.3	58.9 ± 18.6	
Median (ITQ)	59.0 (44–73)	63.0 (48–74)	61.0 (47–74)	
Sex male	733 (59.7%)	1177 (57.2%)	1910 (58.2%)	0.161**
Origin				0.001***
Surgical center	809 (65.9%)	1312 (63.8%)	2121 (64.6%)	
Emergency	289 (23.6%)	588 (28.6%)	877 (26.7%)	
Ward	128 (10.4%)	152 (7.4%)	280 (8.5%)	
Other hospital	1 (0.1%)	4 (0.2%)	5 (0.2%)	
Chronic disease	235 (19.2%)	280 (13.6%)	2768 (15.7%)	0.001**
Admission type				
Clinical	385 (31.4%)	721 (35.1%)	1106 (33.7%)	0.030**
PO urgent	399 (32.5%)	543 (26.4%)	942 (28.7%)	< 0.001**
PO elective	426 (34.7%)	784 (38.1%)	1210 (36.9%)	0.500**
IKA on admission	329 (26.8%)	750 (36.5%)	1079 (32.9%)	< 0.001**
MV on admission	656 (53.5%)	1166 (56.7%)	1822 (55.5%)	0.070**
Sepsis on admission	567 (46.2%)	1039 (50.5%)	1606 (48.9%)	0.016**
VAD	525 (42.8%)	850 (41.3%)	1375 (41.9%)	0.417**
APACHE II				< 0.001*
Mean ± SD	19.5 ± 9.9	22.1 ± 10.3	21.2 ± 10.2	
Median (ITQ)	18.0 (12–26)	21.0 (14–29)	20.0 (13–28)	
SOFA				0.325*
Mean ± SD	7.0 ± 4.7	7.2 ± 4.6	7.1 ± 4.7	
Median (ITQ)	6.0 (3–11)	7.0 (3–11)	7.0 (3–11)	
TISS 28				< 0.001*
Mean ± SD	26.9 ± 7.4	29.5 ± 10.4	28.5 ± 9.5	
Median (ITQ)	27.0 (21–32)	28.0 (21–36)	28.0 (21–34)	
Length of ICU stay				0.060*
Mean ± SD	9.6 ± 16.0	8.5 ± 11.8	8.9 ± 13.5	
Median (ITQ)	4.00 (1–12)	4.0 (1–11)	4.0 (1–11)	
Length of hospital stay				0.117*
Mean ± SD	24.2 ± 28.0	22.8 ± 27.2	23.3 ± 27.5	
Median (ITQ)	17.0 (8–31)	16.0 (8–28)	16.0 (8–29)	
ICU death	453 (36.9%)	674 (32.8%)	1127 (34.3%)	0.016**
Hospital death	587 (47.8%)	908 (44.2%)	1495 (45.5%)	0.041**

*SD* standard deviation, *ITQ* interquartile range, *PO* postoperative, *AKI* acute kidney injury, *MV* mechanical ventilation, *VAD* vasoactive drug, *APACHE* acute physiology and chronic health evaluation, *SOFA* sequential organ failure assessment, *TISS* therapeutic intervention scoring system, *ICU* intensive care unit.

*Mann–Whitney test; **Chi-squared test; ***Fisher's exact test.

The need for invasive mechanical ventilation and dialysis was similar across the study periods. Single vasoactive drug use on ICU admission was more frequent in the second study period, while multiple vasoactive drug use was more frequent in the first study period. An increase in the number of tracheostomies was observed in period 2 of the study (Table 2). There was an increase in the number of admissions over the study period and an increase in the proportion of medical and postoperative admissions for elective surgeries (Table 3).

**Table 2:** Comparison of the use of therapeutic interventions according to the study period.

Variable	Period 1	Period 2	Total	*p* value
MV on admission	755 (61.5%)	1251 (60.8%)	2006 (61.1%)	0.697**
MV Days				0.189*
Mean ± SD	12.3 ± 15.5	11.1 ± 11.8	11.5 ± 13.4	
Median (ITQ)	6.0 (3–17)	7.0 (3–14)	7.0 (3–15)	
Dialysis	252 (20.5%)	462 (22.5%)	714 (21.7%)	0.194**
Dialysis Days				0.267*
Mean ± SD	10.3 ± 21.4	8.8 ± 13.0	9.4 ± 16.5	
Median (ITQ)	6.0 (1–13)	4.0 (1–11)	4.0 (1–12)	
ICP	51 (4.2%)	86 (4.2%)	137 (4.2%)	0.971**
CVC	960 (78.2)	1.581 (76.9%)	2.541 (77.4%)	0.374**
Enteral	648 (52.8%)	1.154 (56.1%)	1.802 (54.9%)	0.065**
Enteral days				0.346*
Mean ± SD	12.8 ± 19.1	11.6 ± 13.3	12.0 ± 15.6	
Median (ITQ)	8.0 (3–16)	8.0 (3–16)	8.0 (3–16)	
PN	80 (6.5%)	63 (3.1%)	143 (4.4%)	< 0.001**
PN Days				0.328*
Mean ± SD	11.7 ± 10.8	10.3 ± 10.8	11.1 ± 10.8	
Median (ITQ)	9.0 (3–16.5)	7.0 (3–14)	8.0 (3–15)	
Single VAD on admission	293 (23.9%)	903 (43.9%)	1.196 (36.4%)	< 0.001**
Multiple VAD on admission	464 (37.8%)	643 (31.3%)	1.107 (33.7%)	< 0.001**
Indwelling arterial catheter	518 (42.2%)	796 (38.7%)	1.314 (40.0%)	0.048**
Pulmonary artery catheter	15 (1.2%)	8 (0.4%)	23 (0.7%)	0.006**
Tracheotomy	205 (16.7%)	407 (19.8%)	612 (18.6%)	0.028**

*MV* mechanical ventilation, *SD* standard deviation, *ITQ* interquartile range, *ICP* intracranial pressure, *CVC* central venous catheter, *PN* parenteral nutrition, *VAD* vasoactive drug.

*Mann–Whitney test; **Chi-squared test.

**Table 3:** Evolution of the number and type of admissions by year of study.

	2013	2014	2015	2016	2017	*p* value*
N. admissions	628 (19.1%)	566 (17.2%)	548 (16.7%)	717 (21.8%)	824 (25.1%)	
Medical	189 (30.1%)	180 (31.8%)	204 (37.2%)	249 (34.7%)	284 (34.5%)	0.054
PO urgent	198 (31.5%)	191 (33.7%)	158 (28.8%)	181 (25.2%)	214 (26.0%)	< 0.001
PO elective	228 (36.3%)	191 (33.7%)	181 (33.0%)	287 (40.0%)	323 (39.2%)	0.033

*N* number, *PO* postoperative.

*Chi-squared linear trend.

**Figure 1:**
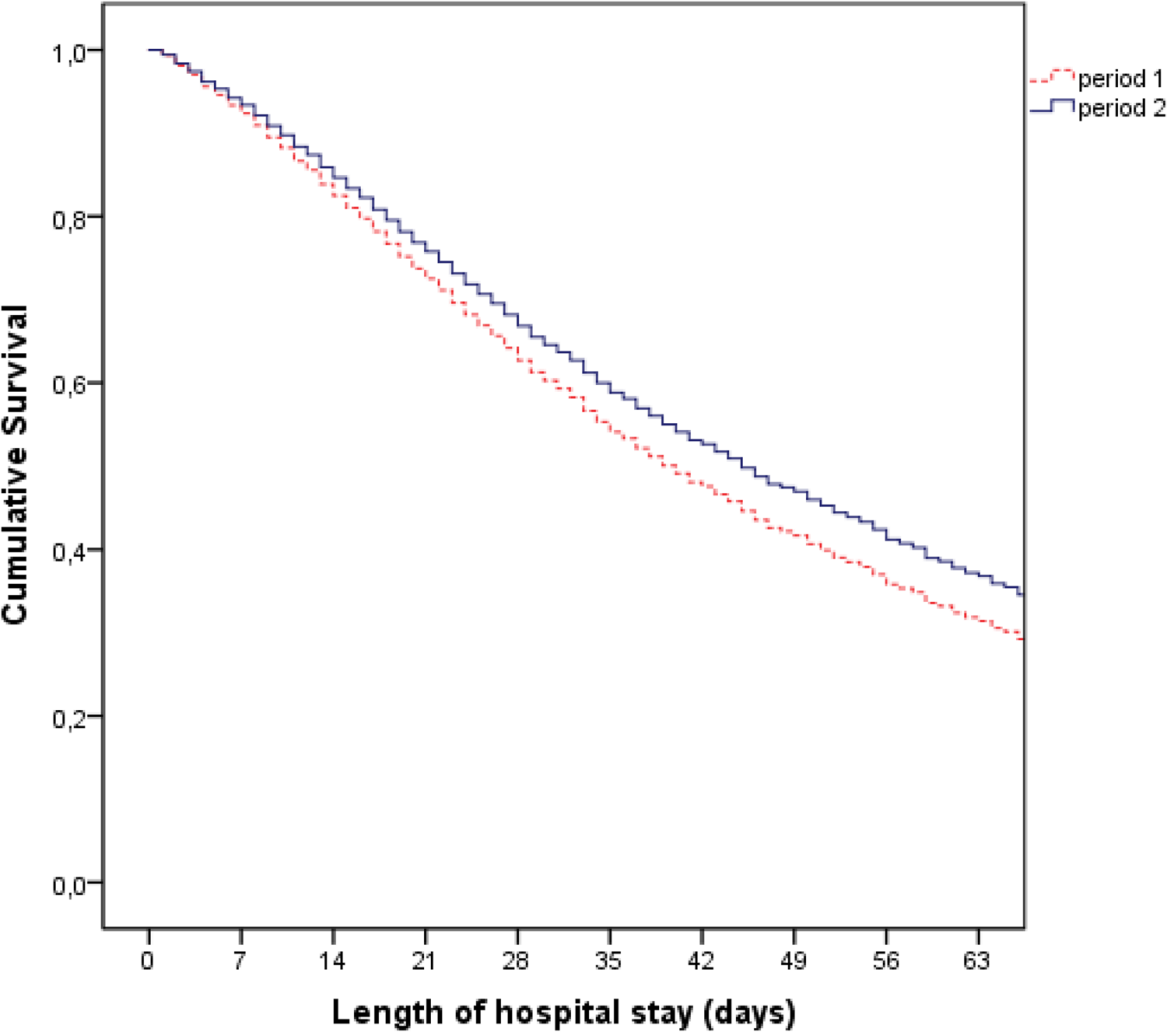
Cox regression survival curve for the hospital outcome according to the screening period by chronological criteria and by prioritization criteria, during the first 60 days of the study. Legend: period 1 = chronologic triage; period 2 = priority triage.

Table 4 presents the Cox regression with the independent variables for the outcome of death at hospital discharge. The risk factors for death were age and APACHE II, SOFA, and TISS 28 scores. Period 2 of the study, when the prioritization triage model was implemented, was a protective factor for hospital death. The survival curve for the death at hospital discharge outcome shows a reduction in patient mortality in study period 2 (Fig. 1).

**Table 4:** COX regression for hospital outcome.

Variable	Hazard ratio	95% CI	*p* value
Age	1.015	1.011–1.018	< 0.001
Male sex	0.881	0.794–0.979	0.018
Chronic disease	1.084	0.952–1.234	0.225
Study period 2	0.870	0.780–0.971	0.013
APACHE II	1.034	1.026–1.042	< 0.001
SOFA	1.085	1.066–1.105	< 0.001
TISS 28	1.008	1.002–1.015	0.011

*APACHE* acute physiology and chronic health evaluation, *SOFA* sequential organ failure assessment, *TISS* therapeutic intervention scoring system, *95% CI* 95% confidence interval.

## Discussion

The present study analyzes the performance of an intensive care unit by comparing two study periods separated by the change in the triage model for patient admission. The change from a chronological model to a prioritization model resulted in a change in the clinical characteristics of patients, an increase in the severity score and in the score of therapeutic interventions, a reduction in the hospital mortality rate of patients and, at the same time, a tendency to a reduction in the length of stay.

The decision on how to triage patients for admission to intensive care units remains a challenging topic. In general, the application of triage for patient care aims to save as many people as possible in times of restriction in the provision of care. Decisions to how to perform triage can be made based on therapeutic benefit, choosing the best candidates for available beds, or on a chronological basis, with the premise that the first to arrive will be the first to be served^12–16^. High-income countries more commonly use score-based screening, such as NEWS2^17^ to decide about ICU admissions. In the present study, we describe a scenario of low- and middle-income countries where the triage system should maximize the benefits obtained from ICU resources available for the community^18^.

In ideal situations, where specialized intensive care beds are available, applying chronological criteria for patient admission seems to be a viable option. However, with increasing demand for beds without the corresponding supply, triage decisions need to prioritize patients who will benefit from this specialized care^5,19^. The occurrence of incompatibility between demand and availability of beds can be frequent in low and middle-income countries. In addition, this scarcity of resources can also occur in extreme scenarios of pandemics or major catastrophes. To assist in the decision process about how to triage patients for care in resource-constrained situations, several protocols and triage tools have been developed^20–25^ however, some real-life experiences have failed to document proper prioritization with the tools developed^22,24^.

Decisions about triaging patients can generate anxiety in responsible professionals. Doubts about personal and family risks, system and patient limitations, lack of experience, and work overload are feelings that permeate these decisions. Therefore, it is recommended that the professional responsible for triage decisions is not from the same team that will be responsible for patient discharge decisions^26^. These teams must have specific training for these functions and need institutional support with adequate maintenance of services in addition to legal support during this process.

Over the years of observation, there was a significant increase in the demand for places for intensive care beds in the institution where the present research was carried out, and this fact motivated the change in the triage model. With this change in triaging, it was possible to observe that there was no significant clinical impact on the mean age of patients admitted to the ICU, with a mean increase of two years of age in the second period. Thus, the prioritization model did not result in restricting access of older adults to intensive care beds. The admission of older adults to the ICU is a reason for discussion in triage models, as there is an aging population that increases the demand for beds by older adults, in addition to the concern of ensuring the admission of older patients who will benefit from treatment. In general, the oldest old tend to receive the same intensity of treatment and have a similar prognosis as older adults^27^.

After changing the triage model in the present study, a greater severity of disease was also observed on admission to the ICU, with a similar degree of organ dysfunction, greater intensity of therapeutic intervention, and, at the same time, a shorter ICU stay and reduced mortality. These findings suggest an improvement in the performance of the intensive care unit, which started to admit more severe patients, but demonstrated better outcomes. Based on these findings, it is possible to speculate that the new prioritization model resulted in improved triage for patients with greater chances of recovery. Within the scenario of high demand for beds, this prioritization model probably resulted in a shorter waiting time for admission to the ICU and faster recovery of admitted patients. The prioritization model resulted in a protection factor with a 13% relative reduction in the chance of death at hospital discharge in the present study.

The reduction of one day in the length of stay in the ICU means an increase in the availability of beds^28^ and this was reflected in a greater number of admissions over the years of the study. The finding that males were a protective factor for the outcome of death at hospital discharge is contradictory to other reports in the literature^29,30^, except in cases of major burns in which the female patient has a worse prognosis^31^.

A limitation of this study is that it was carried out in a single center, which limits the generalization of the results. The retrospective design also brings concerns about causality, which cannot be established from this methodology. The results of survival in intensive care unit study must be interpreted with caution since we excluded patients with priority 5 in the second period and this may explain the higher survival rate. The number of observations limited the possibility of performing an interrupted time series analysis that would have been more effective at dealing with bias due to secular changes. The design and analysis presented in this manuscript can be considered exploratory and support future studies to confirm our findings. A cluster randomized clinical trial would be a design with the best potential to validate the hypothesis. This study also lacks information about the outcomes of the patients who were not admitted to the ICU during the study periods. This issue may be addressed in future research to better understand the impact of the triage models. However, the fact that our results demonstrate the possibility of improving the performance of complex units such as intensive care units with readjustments of triage methodologies and organization of internal flows justifies the investment in further studies on the subject.

## Conclusions

In conclusion, the shift in critically ill patient triage from a chronological model to a prioritization model resulted in improved performance of an intensive care unit, an increase in the number of admissions, a reduction in the length of stay in the ICU and hospital, as well as a reduction in the hospital mortality rate.

## Data Availability

The data of this manuscript will be available from the corresponding author by reasonable request.
